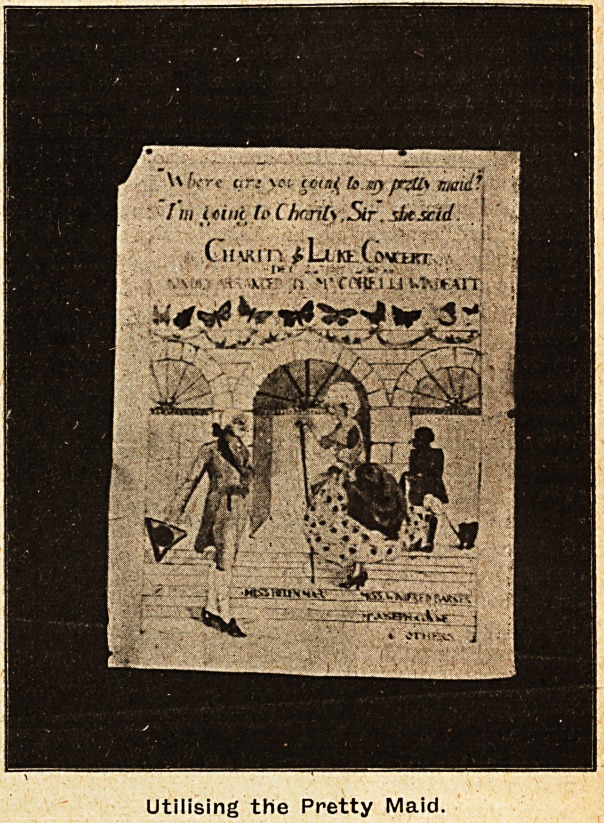# The Best of the Old Year

**Published:** 1918-01-05

**Authors:** 


### ALL OUR HOSPITALS IN UNITY.

### The Best of the Old Year.

We are indeed grateful for the unity of all types of hospitals which have responded with an outpouring of welcome and personal service to our clarion call to provide that Christmas 1917, and the days which followed, should endure in the memory of every warrior, every civil adult and child patient throughout the country. Splendid is the only fitting word to use in describing the united efforts of everybody concerned who has secured for the sick inmates of our hospitals what has been described by some of our fighting men from the Homeland and the Empire as the happiest, merriest, most perfect Christmas Day of their lives. Indeed, some declared they never expected to have such a day in a hospital. It was just "Blighty" at its very best. The same feeling was universally experienced, we are glad to hear, by every hospital resident, including all grades, who gave of their best to make the end of 1917 memorable and fruitful for good, and put forth all they could offer in personal service with merry, thankful hearts.

Some Mandarins in Lydia.

These celebrations will be memorable too in the history of the Nation, because for the first time members of the Royal Family in the persons of our beloved Queen Alexandra and her devoted and much honoured daughter, H.R.H. Princess Victoria, gave up an afternoon that they might pass it in the London Hospital, of which Her Majesty is the President, with 800 "air-raid children" who were assembled in the large waiting-hall of the out-patient department, where Father Christmas, Charlie Chaplin, Little Bo-peep, the "Glaxo" baby, Punch and Judy, and his other attendants, dressed to represent every nationality known to children and some others, devoted themselves to the amusement of the little folk present. Queen Alexandra first visited the children who were having tea, where she entered heartily into the spirit of the occasion, pulled crackers with all and sundry, and gladdened all hearts, not only by her presence but by her cordiality and evident affection for the little ones, several of whom were still crippled, and others invalids. Tea over, the children were seated so that everyone could enjoy in comfort the special cinematograph. Her Majesty and the Royal party occupied chairs in the balcony, from which they had a good view of the pictures, and were also able to watch and fully enjoy the merry children full of fun and pleasurable excitement. We are glad to know that Queen Alexandra and Princess Victoria spent one of the most enjoyable afternoons of the year in the East End with 800 little sufferers from air-raid inhumanity. All honour to these Royal and Illustrious Ladies. Also to Mr. E. W. Morris, the House Governor, and the *Daily Mail*, whose combined action secured the necessary means and supplied the organising power to accomplish these memorable results.

But the enthusiastic self-denial and genuine interest displayed throughout our hospitals this year has revealed amongst other things the notable development in the type of women who are being attracted to nurse-training schools. The decorations in many wards demonstrably prove this fact, for their artistic design and form this year were far and away better and, if possible, more attractive than anything hospital wards have contained in the past. Space would not permit, if it were even possible, to embrace every type of decoration to be met with in the hospitals this year. We can only offer a few of special excellence which it was our privilege to examine and enjoy.

First it should be understood that at Guy's Hospital, which has ever been foremost as the home of artistic decorations at Christmas, so great was the interest that several of the residents with artistic gifts devoted many evenings in preceding weeks this year to designing programmes of the concerts to be given in the various wards. Some of the results will be found in the illustrations we have given.

**ALL OUR HOSPITALS IN UNITY—** (*concluded*).

They show such marked talent and considerable variety that we wish it were possible to publish the whole of them, for they formed quite a gallery in the Quad of the hospital during the days embraced by the entertainments. Some of the sisters and nurses, too, exhibited much skill and rare gifts in design and decoration. This was notably the case

A 1917 Lamp Shade.

in the matter of lamp-shades, which had to be used throughout the hospital owing to the impossibility of employing the myriad of small electric lights which had formed such an attractive feature on several occasions during recent years. It is just to say, however, that, thanks to the infinite variety of designs, and some most excellent drawings upon silk, Japanese paper, and other materials used for the purpose, the general effect of the wards on Christmas Day 1917 presented a beauty and infinite variety of charm which in our view made the effect in many wards more attractive than ever. These changes and the exhibition of the ability of such an army of helpers were infinitely cheering to everyone who has followed closely the development of the modern hospital in these Islands, for they testify to the presence on the nursing staff of women of great natural capacity and many talents. Further, for the twentieth year at least the out-patient department at Guy's afforded an afternoon of continuous rejoicing and happiness to out-patient children, and all who had the privilege to be present and take part in this joyous merry-making and the inspiriting scenes it provided.

Another point worthy of mention was the quiet whole-hearted enjoyment of everybody everywhere within the hospitals this year. War time had necessarily done much to depress many, but the outpouring of personal service cheered the hearts of the workers, who were grateful for the opportunity to spend the days of seasonable rejoicings this year by absorbing themselves in the successful effort to make every sick person forgetful of their own ailments and contentedly happy. Indeed, we incline to the belief that the loving energy and effort so successfully expended by the staff—the sisters and nurses—and the patients in making the wards attractive and beautiful—the latter of whom enjoyed themselves immensely—resulted in making for the residents in the wards this year an atmosphere wholly for the good and inspiration of every inmate.

We have written thus fully, and are giving a number of illustrations, in the hope of encouraging every hospital to work on the lines described, and to afford some idea of the methods and results which can be achieved by ability and devotion without incurring any serious outlay. The word outlay reminds us, and we record the fact with gratitude, that in 1917, for the first year, owing no doubt to the special appeal we made to members of the committees and governors of our hospitals to provide the necessary funds to pay all the costs

A Typical Programme.

entailed, no sister—in some of our largest hospitals at least, and we have reason to believe it is generally so—was placed under the necessity to spend her own money, because the funds given for the purpose were more than ample to defray the total cost of the year's decorations and amusements.

**ALL OUR HOSPITALS IN UNITY**—(*continued*).

It may be well to add that success, so far as the decoration of a hospital ward is concerned, depends largely upon an arrangement between the sisters that every ward shall have its own scheme, which shall be distinct in character. Among an infinite variety of designs, we may mention the Astley Cooper Ward, where the subject was winter, including a village wrapped in snow, the church with its Christmas decorations, and every cottage snowed up. In Luke a Chinese design was introduced with some extraordinary zoological types to be found amongst the decorations at the head of many of the beds. Here, too, the design for lamp shades was original and telling. In Cornelius, owls and moons, the lamp shades giving the effect of moonlight in the evening, was welcome. Martha described herself as japonica, and the most was made of Japanese art. Job aimed at warlike simplicity in decoration, and made effective use of dragon flies of all colours and varieties. Infinite skill was exhibited in Lazarus, where the aim was to give the effect of a pack of cards. Naaman adopted sunflowers, and the lamp shades were so varied as to fix the attention of the artist. Lydia gave an Eastern scene with mandarins in full costume, and revealed a surprising ability by the combined effort of those responsible.

Please Interpret the Owl and his Surroundings.

Charity was a scene to rejoice the heart of all butterflies, for the butterfly was the underlying feature of the decorations. Dorcas also used butterflies with flags and Japanese lanterns. The love of flowers provided a striking exhibition of colour by the use of violets in Patience and Samaritan. "Eyes" Ward broke out into poppies and corn with dragon flies and an atmosphere of summer. Laburnum and wisteria made the decorations in Clinical effective. Mary utilised Father Christmas and trees to provide a Christmas scene. Addison was ambitious, aiming at a unique winter sky in 1917, with aircraft, roofs, cats and chimney-pots. Esther very properly selected sweet peas with many

Utilising the Pretty Maid.

shades of colour. Queen disguised herself by the introduction of an excellent assortment of Japanese lanterns. Bright took itself to the moorlands, and contained witches and brooms. Eveline, gaining the aid of a lover of flowers, contained nasturtiums in gilt baskets, that lent themselves to beautiful effects in artificial light.

There remain the play-bills. Here is one with Tommy standing sentry in full kit with the words "There is a War on, but come to Astley Cooper on December 31, and forget the War, from 8 to 10 p.m." Another had for centrepiece an excellently drawn owl with the crescent, and the words "Marvelious Attraction," being Stars, Knuts, a doe, and Knuts. There was an excellent drawing of a town crier with his bell and the programme of the concert, and another of the entrance-steps going up to the Quad, from the Park, with a cavalier in sixteenth-century clothes, and a lady accompanied by her black attendant in old-time costume, with the words: "Where are you going to, my pretty maid?' 'I am going to Charity, Sir, she said."—"Charity and Luke Concerts," There are many more drawings, all excellent, showing infinite ingenuity and a fund of humour, but alas! our space is exhausted.

## Figures and Tables

**Figure f1:**
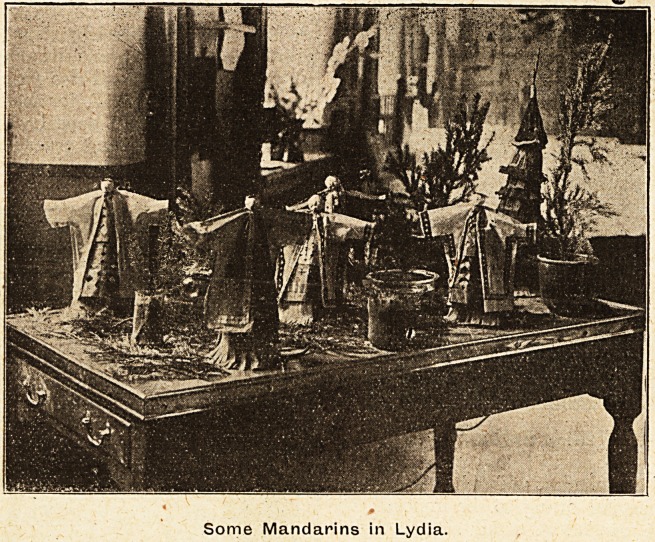


**Figure f2:**
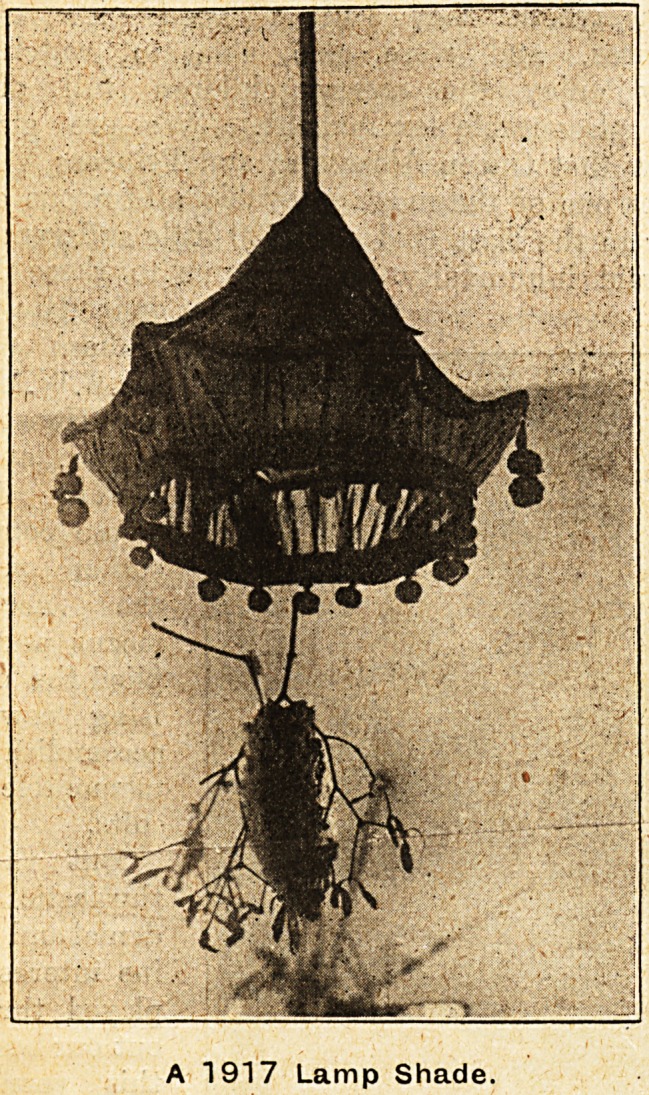


**Figure f3:**
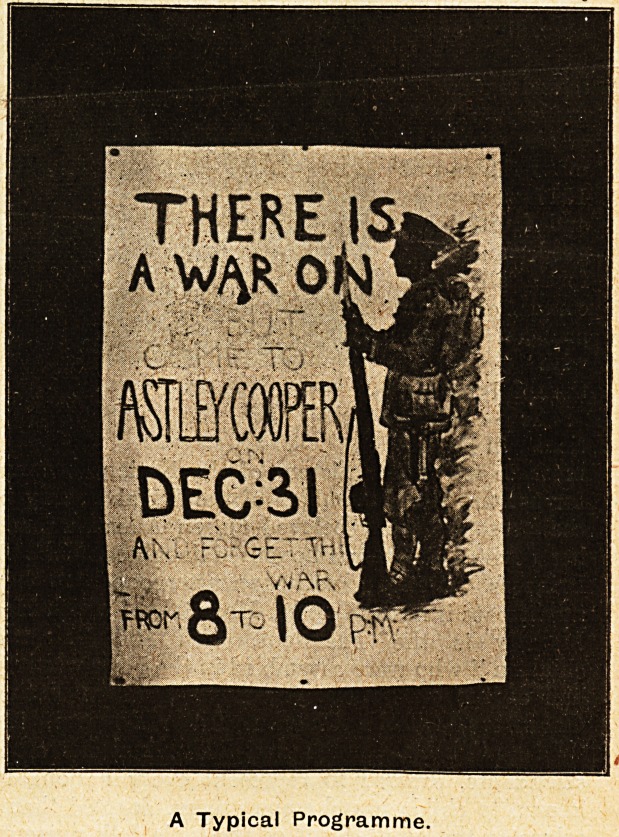


**Figure f4:**
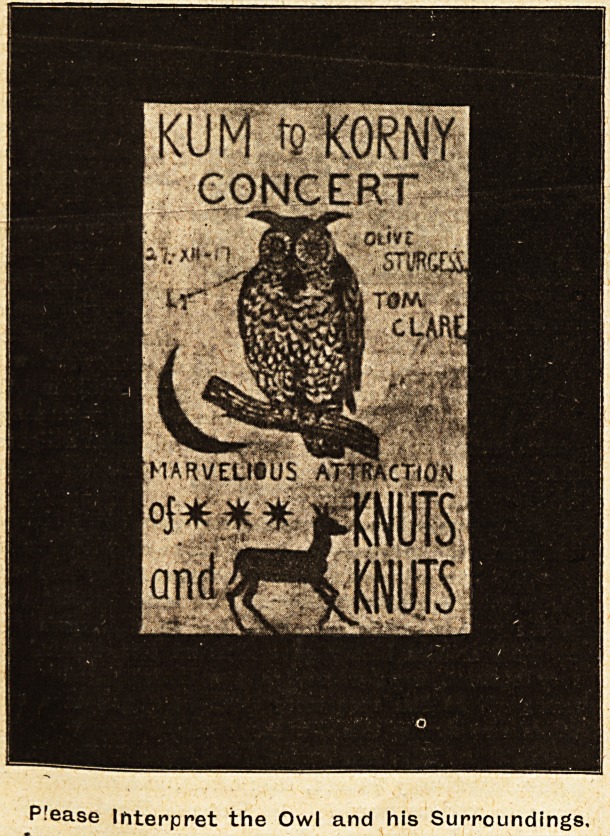


**Figure f5:**